# Characterization of METTL16 as a cytoplasmic RNA binding protein

**DOI:** 10.1371/journal.pone.0227647

**Published:** 2020-01-15

**Authors:** Daniel J. Nance, Emily R. Satterwhite, Brinda Bhaskar, Sway Misra, Kristen R. Carraway, Kyle D. Mansfield

**Affiliations:** Department of Biochemistry and Molecular Biology, Brody School of Medicine, East Carolina University, Greenville, North Carolina, United States of America; University of Toronto, CANADA

## Abstract

mRNA modification by N6-methyladenosine (m6A) is involved in many post-transcriptional regulation processes including mRNA stability, splicing and promotion of translation. Accordingly, the recently identified mRNA methylation complex containing METTL3, METTL14, and WTAP has been the subject of intense study. However, METTL16 (METT10D) has also been identified as an RNA m6A methyltransferase that can methylate both coding and noncoding RNAs, but its biological role remains unclear. While global studies have identified many potential RNA targets of METTL16, only a handful, including the long noncoding RNA MALAT1, the snRNA U6, as well as the mRNA MAT2A have been verified and/or studied to any great extent. In this study we identified/verified METTL16 targets by immunoprecipitation of both endogenous as well as exogenous FLAG-tagged protein. Interestingly, exogenously overexpressed METTL16 differed from the endogenous protein in its relative affinity for RNA targets which prompted us to investigate METTL16's localization within the cell. Surprisingly, biochemical fractionation revealed that a majority of METTL16 protein resides in the cytoplasm of a number of cells. Furthermore, siRNA knockdown of METTL16 resulted in expression changes of a few mRNA targets suggesting that METTL16 may play a role in regulating gene expression. Thus, while METTL16 has been reported to be a nuclear protein, our findings suggest that METTL16 is also a cytoplasmic methyltransferase that may alter its RNA binding preferences depending on its cellular localization. Future studies will seek to confirm differences between cytoplasmic and nuclear RNA targets in addition to exploring the physiological role of METTL16 through long-term knockdown.

## Introduction

Methylation on the sixth position of the base moiety of adenosine (m6A) is one of the most common mRNA modifications in eukaryotes, and it has been shown to affect all aspects of post-transcriptional regulation including mRNA splicing, stability, and translation [1–9]. Methyltransferase like -3 and -14 (METTL3 and METTL14) and Wilms’ tumor associating protein (WTAP) in addition to KIAA1429 are all components of the mRNA m6A methyltransferase complex, which uses a S-adenosyl methionine (SAM) binding domain on METTL3 to methylate specific mRNAs for methylation with a RRACH m6A consensus sequence [10–15]. Many RNA binding proteins (RBPs) including the YTH family of proteins modulate the effects of m6A through specific binding to the methylated RNA. For example, YTHDF1 has been shown to increase translation of m6A containing mRNA, while YTHDF2 appears to direct mRNA degradation and YTHDF3 appears to play roles in both processes [5–8, 16, 17]. m6A has been shown to play a role in a number of physiological processes including embryonic stem cell differentiation, circadian rhythms, response to hypoxia and other stressors, and is implicated in many different aspects of cancer [1, 9, 16, 18–27].

METTL16 has also been identified as an RNA m6A methyltransferase that methylates both coding and noncoding RNAs. Primarily, METTL16 has been shown to methylate the U6 snRNA [28, 29]. It can also bind and methylate the long noncoding RNAs MALAT1 and XIST [28, 30]. In addition, METTL16 has been shown to bind and methylate mRNAs, including MAT2A, which can regulate its alternative splicing in response to cellular SAM levels [29, 31, 32]. Furthermore, global analysis suggests that many other mRNAs including RBM3 and STUB1 may also be METTL16 targets [28].

Perhaps the most intriguing aspect of the METTL16 methyltransferase is the importance of structure when binding targets, not just sequence like the METTL3/METTL14/WTAP complex. METTL16 m6A methylation of MAT2A is reliant upon a conserved hairpin (hp1) for binding and a similar sequence and structure is required for U6 methylation as well, but interestingly, is not readily apparent in other METTL16 targets [29]. In *in vitro* methylation studies, METTL16 appears to prefer stem loop structures with the methylated adenosine being unpaired in a single stranded loop or bulge [31, 33]. Additionally, instead of the heterodimeric “writer” complex formed by METTL3/METTL14/WTAP m6A methyltransferase, METTL16 functions as a homodimer [34]. This homodimeric METTL16 is necessary for binding the MALAT1 triple helix, although monomeric METTL16_291, which contains only the methyltransferase domain, is sufficient for methylating U6 and MAT2A RNAs [29, 34].

At a molecular level, the effects of METTL16 m6A activity are best understood in the context of cellular S-adenosylmethionine (SAM) levels and intron retention of MAT2A pre-mRNA. SAM is a methyl donor for most cellular methylation reactions and is created using SAM synthetases that convert methionine and ATP into SAM [29]. In human cells, the SAM synthetase is encoded by the MAT2A gene and is expressed in all cell types except liver cells. Methionine depletion stabilizes MAT2A mRNA, which has six hairpin structures (hp1-6) in its 3’ UTR that serve as binding sites for METTL16. When intracellular SAM levels are high, METTL16 binds the hp1 of MAT2A RNA, methylates it, and quickly dissociates to support intron retention. Intron retention targets the MAT2A mRNA for nuclear degradation. Low intracellular levels of SAM do not allow for efficient methylation by METTL16, increasing METTL16 occupancy on the mRNA which results in increased splicing of the retained intron. This increases stabilization and translation of the MAT2A mRNA and production of SAM synthetase which increases SAM levels in the cell. Additionally, the YTHDC1 m6A “reader” protein may play a role in processing the mature MAT2A mRNA and monitoring intracellular SAM levels [32].

Other than regulating MAT2A in response to SAM levels, the physiological significance of METTL16 is largely unknown at this point. METTL16 does appear to be vital for the proliferation/survival of a number of cancer cells [35] and has been identified in a number of screens for essential genes [36–40]. Interestingly, whole mouse METTL16 knockout results in blastocysts that are unfit to develop further and abort, although the reason for this is not definitively known, but may also be due to regulation of MAT2A expression [31]. Thus, METTL16 appears to be essential for mammalian life, even though its physiological role has not been fully characterized.

In this study, we have confirmed that METTL16 binds a number of coding and noncoding RNAs and identify a number of novel METTL16 targets. In addition, we show that overexpression of METTL16 can affect both its cellular location as well as its RNA binding preferences. Our results suggest that the majority of METTL16 protein resides in the cytoplasm of a number of cell types, and that knockdown of METTL16 protein can affect the expression of a few of its mRNA binding targets. These results suggest that METTL16 may have additional roles in the cell that could contribute to its essentiality.

## Results

### Identification of METTL16 binding targets

**Fig 1:**
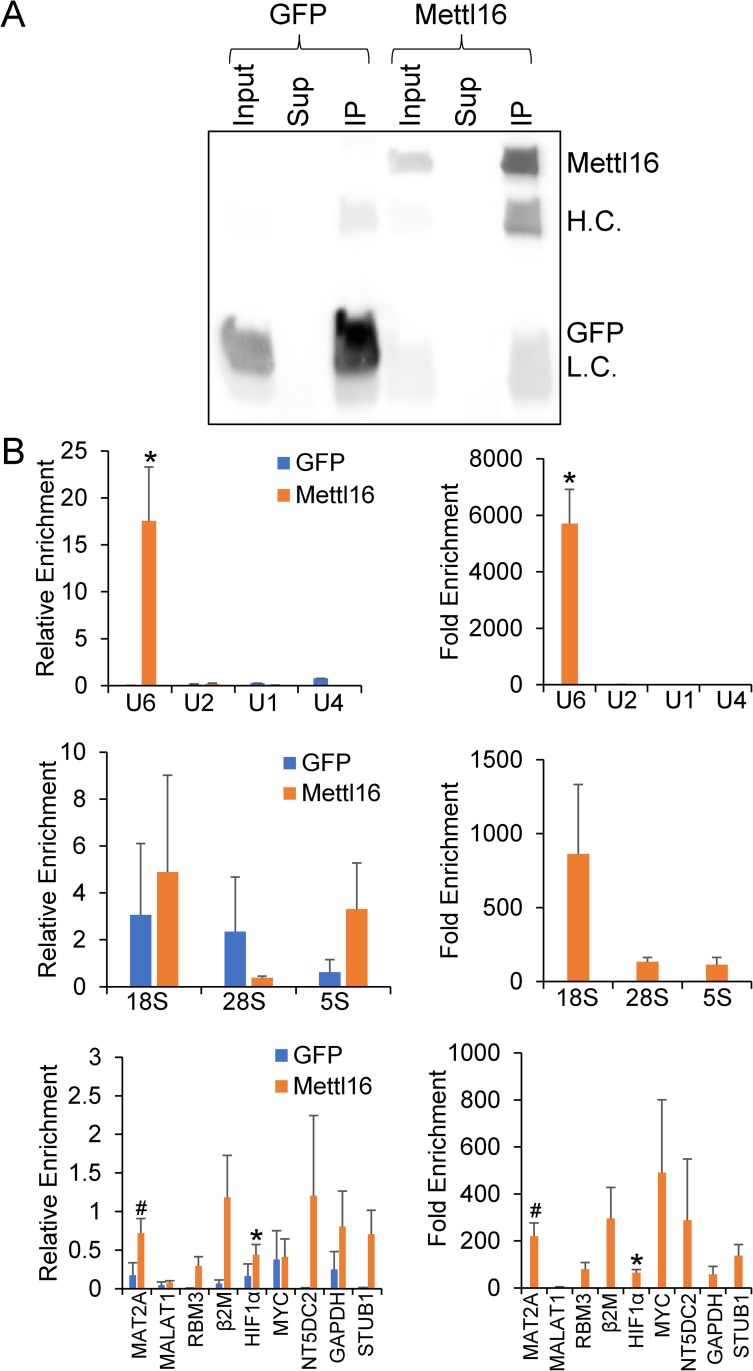
Identification of FLAG-METTL16 targets. FLAG-METTL16 or FLAG-GFP protein was overexpressed and immunoprecipitated from HEK293T cells. (A) Input, Supernatant (Sup), and Immunoprecipitated (IP) protein were subjected to Western blotting to confirm successful immunoprecipitation (Antibody Heavy Chain (H.C.) and Light Chain (L.C) are indicated). (B) Associated RNAs were isolated via Trizol and real-time PCR was used to determine enrichment. The left panel depicts data as relative enrichment compared to the input, while the right panel shows enrichment in the METTL16 relative to the FLAG-GFP control immunoprecipitation. (*) P≤ 0.05, (#) P≤0.1 by paired Student’s t-test. Error bars represent SEM of four to seven experiments.

To identify potential METTL16 RNA targets as well as verify reported literature targets, HEK293T cells were transiently transfected with either FLAG-GFP or FLAG-METTL16 overexpression constructs. Cell extracts were then subjected to Ribonucleoprotein Immunoprecipitation (IP) using FLAG magnetic beads. Western blotting was used to confirm successful immunoprecipitation as evidenced by depletion of the expected band in the supernatant and enrichment in the IP (Fig 1A). RNA was also extracted, and real-time PCR was used to measure enrichment of METTL16 targets. Two methods were used to calculate enrichment. In the first analysis, the amount of the RNA target in the GFP or METTL16 IP was compared to the input levels from 50% of the sample to generate a relative enrichment (Fig 1B, left panels). Specifically, the value is a fold enrichment in the IP relative to the Input and is calculated by raising two to the power of the Cq value of the IP subtracted from the Cq value of the Input. In the second analysis, fold enrichment of the RNA target in the METTL16 IP compared to the GFP IP was calculated (Fig 1B, right panels) by dividing the METTL16 relative enrichment (as described above) by the GFP relative enrichment value. By comparing enrichment to both the input as well as a negative IP we can get a better sense of the nature of the binding and have greater confidence in identifying METTL16 binding targets. As shown in Fig 1B, U6 snRNA appears to be the primary binding target of METTL16 by either analysis. Importantly, U1 and U2 snRNA, which are not known to harbor an m6A, served as negative controls and exhibited very little enrichment in the METTL16 IP. It does appear that 18S rRNA is also a target, showing an almost 1,000-fold enrichment in the METTL16 IP over the GFP IP. Interestingly, in addition to MAT2A, a number of mRNA’s including β2M, MYC, and NT5DC2 also appeared to be FLAG-METTL16 targets, while the long noncoding RNA (lncRNA) MALAT1 did not appear to be bound by FLAG-METTL16.

**Fig 2:**
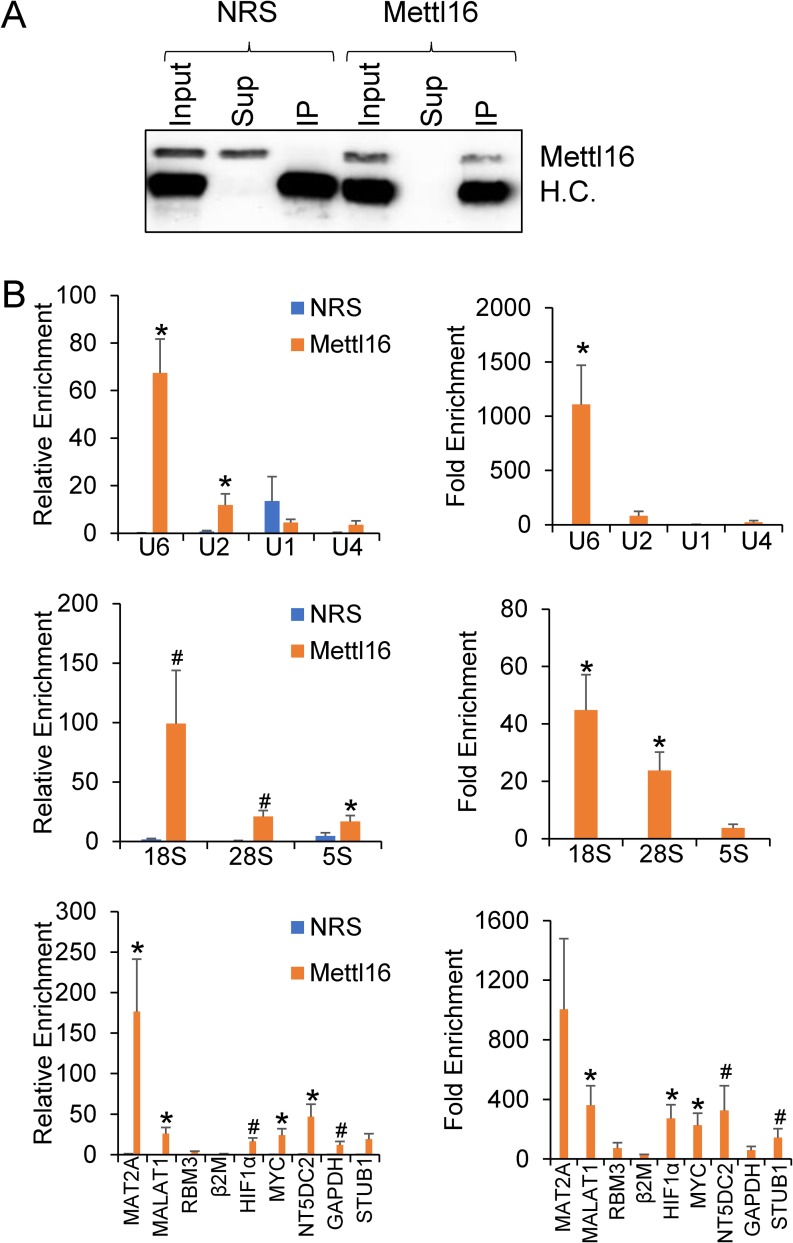
Identification of endogenous METTL16 targets. HEK293T extracts were immunoprecipitated with either METTL16 antibody or normal rabbit serum (NRS) as a negative control. (A) Input, Supernatant (Sup), and Immunoprecipitated (IP) protein were subjected to Western blotting to confirm successful immunoprecipitation (Antibody Heavy Chain (H.C.) is indicated). (B) Associated RNAs were isolated via Trizol and real-time PCR was used to determine enrichment. The left panel depicts data as relative enrichment compared to the input, while the right panel shows enrichment in the METTL16 relative to the FLAG-GFP control immunoprecipitation. (*) P≤ 0.05, (#) P≤0.1 by paired Student’s t-test. Error bars represent SEM of six experiments.

To determine if these targets were the same for endogenous METTL16, we repeated the immunoprecipitation in HEK293T extracts using either METTL16 antibody or normal rabbit serum (NRS) coated magnetic beads. Western blotting confirmed that the IP was successful with METTL16 protein remaining in the supernatant of the negative IP while appearing in the IP lane of the METTL16 antibody (Fig 2A). Real-Time PCR was again used to measure RNA enrichment of targets either compared to the input levels (Fig 2B, left panels) or in relation to the negative IP after normalizing for input levels (Fig 2B, right panels). Interestingly, in contrast to the FLAG-METTL16 immunoprecipitation, MAT2A mRNA appeared to be the primary target of endogenous METTL16, although U6 snRNA was still a significant target. Additional mRNAs such as HIF-1α, MYC, and NT5DC2 were also identified as potential METTL16 targets while 5S rRNA and U1 and U2 snRNA appear to not be bound by METTL16, which is expected given that they are not known to be m6A methylated. In contrast to the FLAG-IP, the lncRNA MALAT1 did appear to be a binding target of the endogenous METTL16.

### Investigation of METTL16 cellular localization

**Fig 3:**
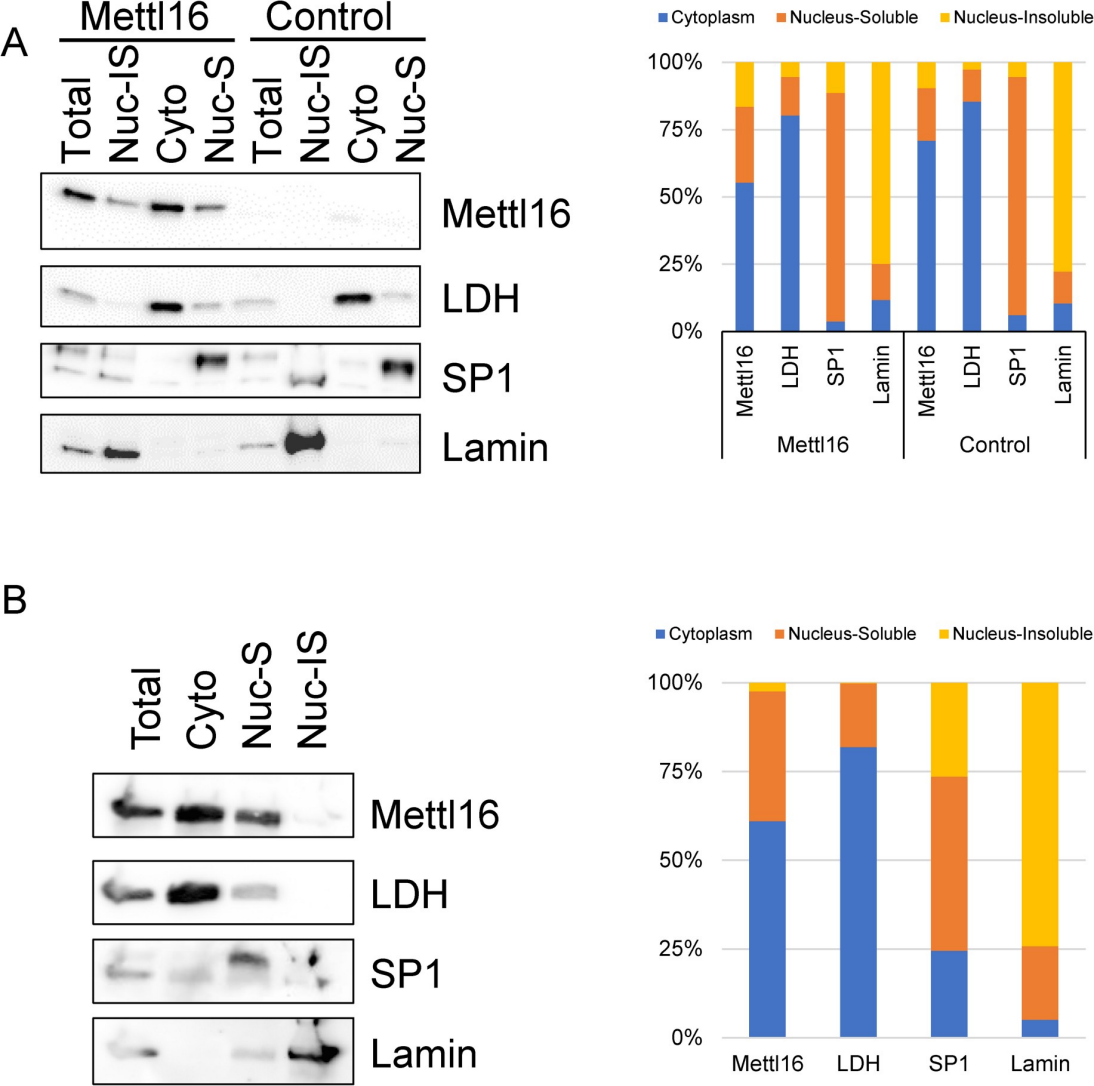
Determination of METTL16 cellular localization in HEK293T cells. (A) Extracts from HEK293T cells stably expressing FLAG-METTL16 or an empty vector control were separated into Total, Insoluble Nuclear (Nuc-IS), Cytoplasmic (Cyto), and Soluble Nuclear (Nuc-S) fractions and subjected to Western blot with METTL16 antibody to determine METTL16 cellular localization. Lamin B was used as an insoluble nuclear marker, SP1 as a soluble nuclear marker, and lactate dehydrogenase (LDH) as a cytoplasmic marker. (B) Biochemical cellular fractionation was also used to determine the location of endogenous METTL16 protein in untreated HEK293T cells. (representative of three to five experiments).

Based on the differences in targets between exogenous FLAG-tagged and endogenous METTL16, we theorized that METTL16 localization may change based on expression levels and that this may impact target selection. Biochemical fractionation of FLAG-METTL16 overexpressing HEK293T cells revealed that exogenously expressed METTL16 protein was found in both the cytoplasmic and nuclear fractions (55% in cytoplasm; 45% in nucleus) (Fig 3A). Lactate Dehydrogenase (LDH) served as a marker for the cytoplasmic fractions, while the nuclear matrix protein Lamin B and the transcription factor Specificity Protein 1 (SP1) served as markers for the insoluble and soluble nuclear fractions respectively and confirm successful fractionation. To investigate whether overexpression affected METTL16 cellular localization we also analyzed endogenous METTL16’s localization in HEK293T cells using biochemical fractionation (Fig 3B). Compared to the FLAG-overexpressed METTL16, slightly more endogenous METTL16 was found in the cytoplasm (60%) relative to the nucleus (40%). These results suggest that overexpression of METTL16 may affect its cellular localization and that this may impact the RNA targets identified in the immunoprecipitations.

Previously, METTL16 has been reported to be a predominantly nuclear protein [28–30, 41]. Using an antibody from a previous study we attempted to use immunohistochemistry to verify METTL16’s cellular localization [30]. Initial experiments showed METTL16 staining in both the cytoplasm and nucleus of HEK293T cells (S1 Fig). To validate antibody specificity, we ran both Western blotting and immunohistochemistry on control and METTL16 siRNA knockdown HEK293T cells. Despite almost complete knockdown of METTL16 protein by siRNA (as confirmed by western blotting) similar staining patterns and intensities were observed in both control and METTL16 siRNA treated samples suggesting that the immunohistochemical staining observed may be non-specific (S1 Fig). A second antibody gave similar results with fluorescent signal in both the cytoplasm and nucleus of HEK293T cells. However, once again, the signal was not decreased in the immunohistochemistry when METTL16 was depleted with long-term (6 day) siRNA treatment suggesting that the majority of the signal from this antibody is also coming from non-specific binding (S2 Fig).

**Fig 4:**
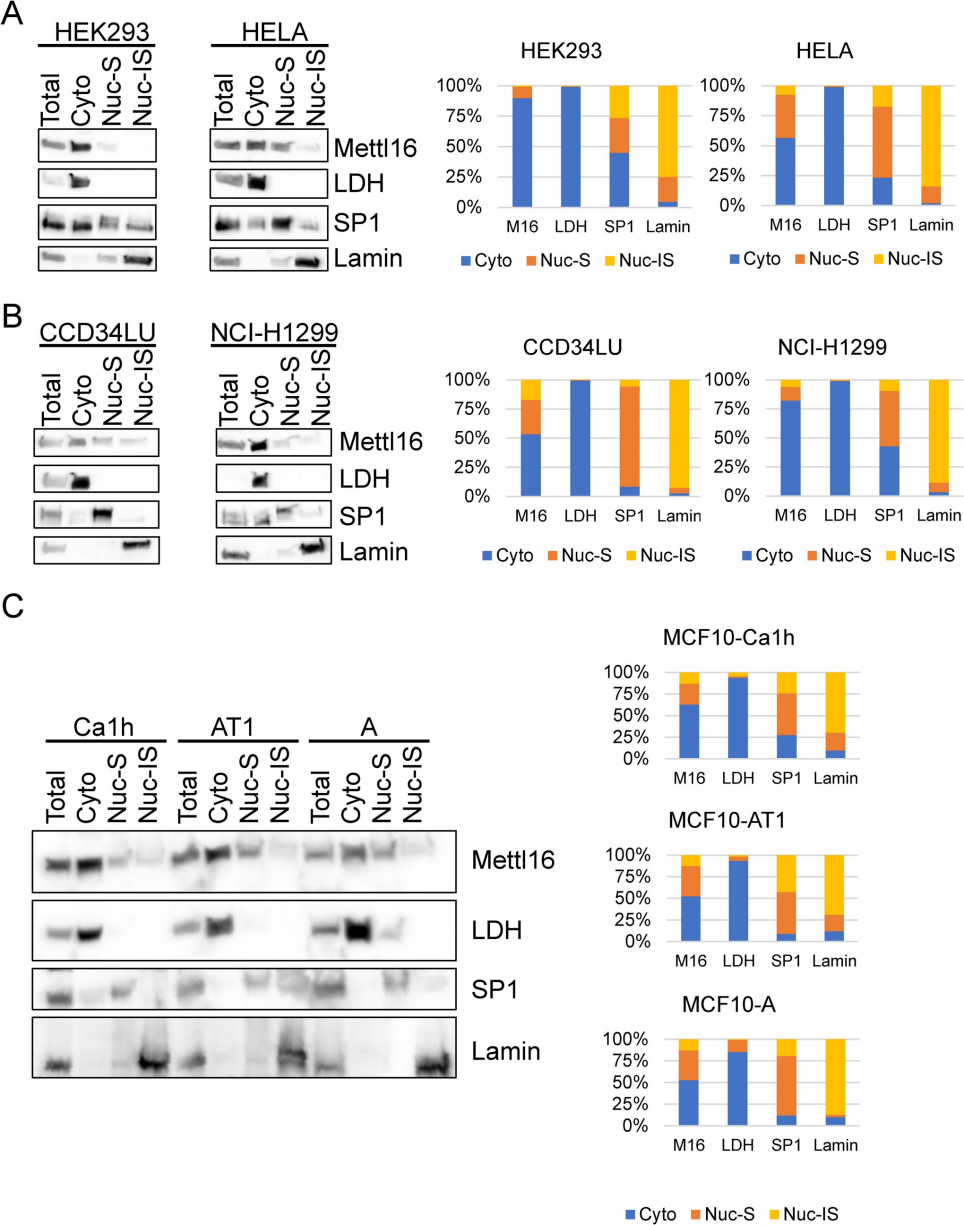
Determination of METTL16 cellular localization in multiple cell lines. (A) Extracts from HEK293 or HELA cells were separated into Total, Cytoplasmic (Cyto), Soluble Nuclear (Nuc-S), and Insoluble Nuclear (Nuc-IS) fractions and subjected to Western blot with METTL16 antibody to determine METTL16 cellular localization. Lamin B was used as an insoluble nuclear marker, SP1 as a soluble nuclear marker, and lactate dehydrogenase (LDH) as a cytoplasmic marker. (B, C) Biochemical cellular fractionation was also used to determine the location of METTL16 protein in lung cell lines CCD34LU and NCI-H1299 as well as breast cancer cell lines MCF10-Ca1h, MCF10-AT1, and MCF10-A. (representative of two to three experiments).

As an alternative approach, we again used biochemical fractionation to examine METTL16’s localization in two other cell lines in which immunohistochemistry had indicated nuclear localization [28, 30]. As shown in Fig 4A, in HEK293 cells, METTL16 protein appears to be predominately located in the cytoplasm with almost 90% of the protein in the cytoplasm, while in the HELA cells approximately 55% of METTL16 protein appears cytoplasmic. To determine if this a more widespread observation, we also examined METTL16’s localization in a lung fibroblast cell line (CCD34LU) as well as a lung cancer cell line (NCI-H1299) and again found METTL16 to be predominant in the cytoplasm of both cell types (Fig 4B). We also examined METTL16’s localization in a series of MCF10 breast cancer cells representing different stages of breast cancer progression. MCF10-A’s represent immortalized, yet non-tumorigenic cells. MCF10-AT1’s are transformed but are weakly tumorigenic, while the MCF10-Ca1h line represents a highly aggressive tumorigenic cell line [42, 43]. Again, we saw at least 50% of METTL16 protein located in the cytoplasm of all three of these cell lines (Fig 4C), confirming that in addition to its reported nuclear localization, METTL16 protein is found in the cytoplasm of a number of cell types and localization does not appear to be affected by transformation.

### Effect of METTL16 knockdown on target expression

**Fig 5:**
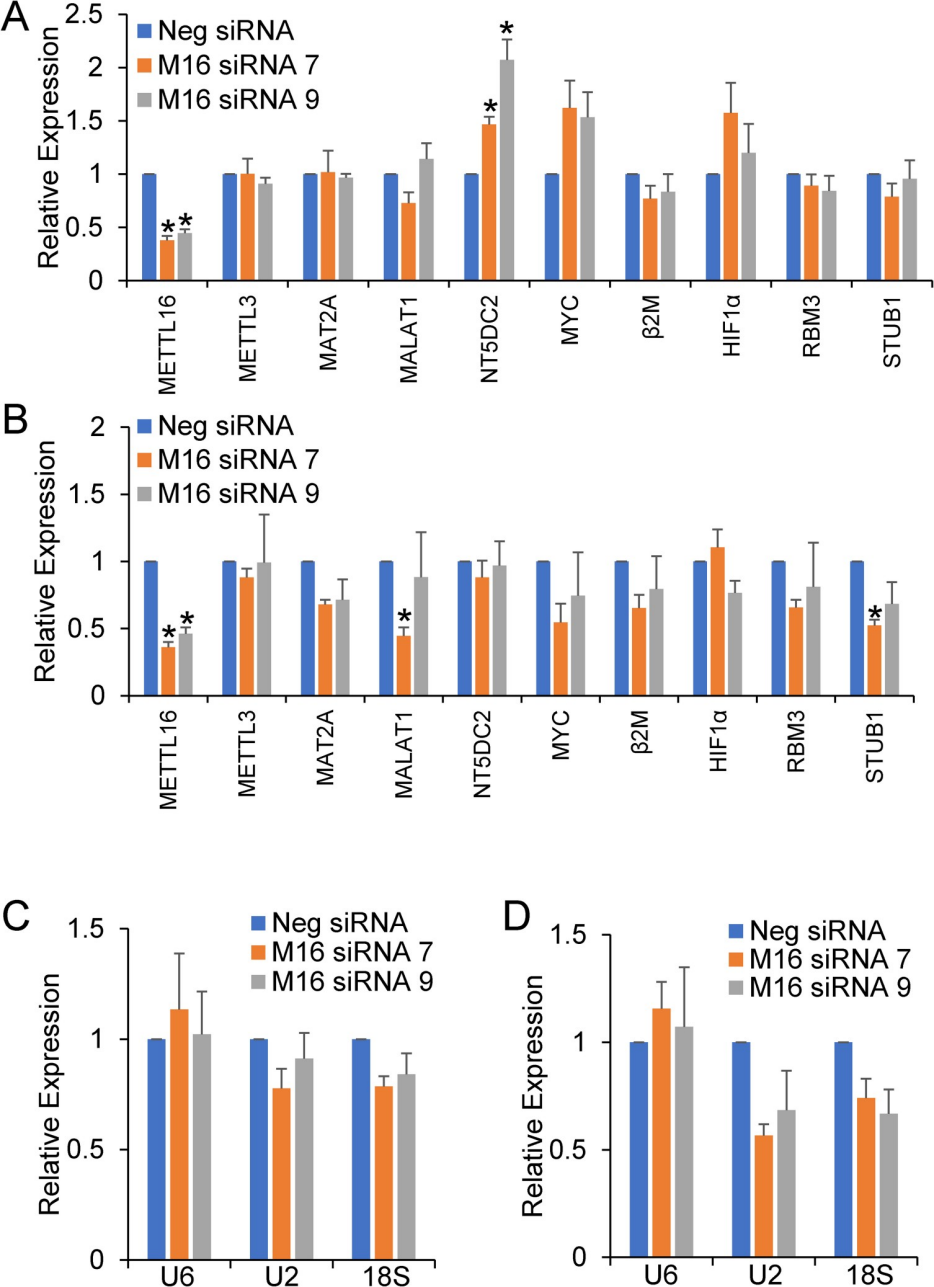
Effect of METTL16 knockdown on mRNA expression. HEK293T cells were treated for 2 days (A, C) or 6 days (B, D) using either a negative control siRNA (Neg) or one of two METTL16-specific (7 & 9) siRNAs. Real-time PCR was used to determine the effect on RNA expression normalized to GAPDH and expressed as relative to the negative siRNA control. (*) P≤ 0.05 by one-way ANOVA with post-hoc Tukey HSD test. Error bars represent SEM of five experiments.

**Fig 6:**
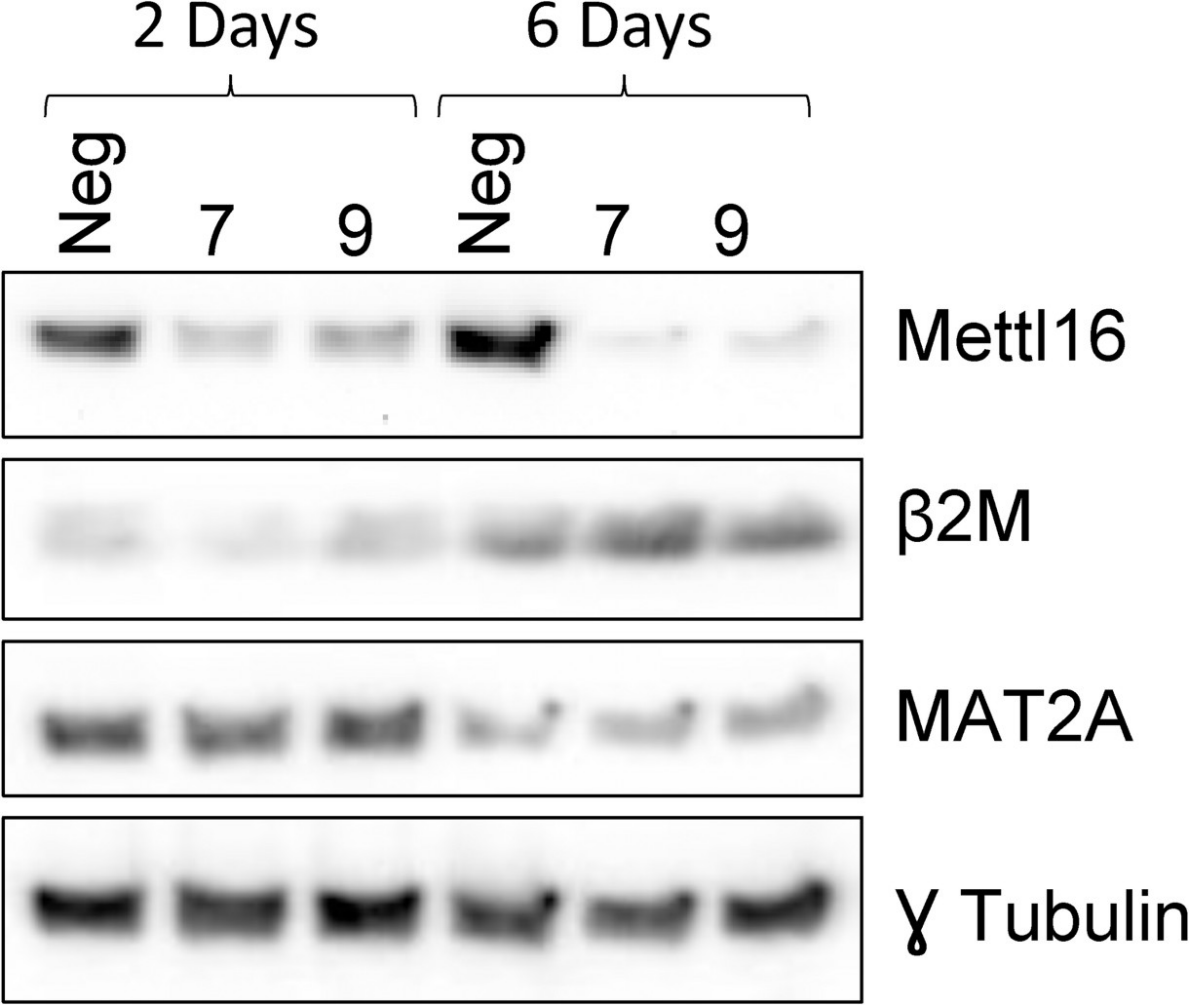
Effect of METTL16 knockdown on protein expression. HEK293T cells were treated for 2 days (A) or 6 days (B) using either a negative control siRNA (Neg) or one of two METTL16-specific (7 & 9) siRNAs. Western blotting was used to determine the effect on protein expression. (representative of five experiments).

We attempted to determine the physiological role of METTL16 by knocking it out using CRISPR, but we were unable to create stable cell lines. This is in line with previous literature reports that suggest that METTL16 is likely an essential gene [29, 36]. METTL16 was instead knocked down for an extended period of time using siRNA. In this set of experiments, HEK293T cells were transfected with either a scrambled negative control siRNA or one of two METTL16-specific siRNAs. After 48 hours of siRNA expression, cells were harvested or replated and retransfected the following day with the same siRNA. Cells were then harvested 6 days after the initial transfection (3 days after the 2^nd^ transfection). Western blotting confirmed that compared to the negative control siRNA, treatment with either METTL16 siRNA resulted in significant knockdown of METTL16 expression at both the RNA (Fig 5A) and protein (Fig 6) level, with 6 days treatment resulting in almost complete loss of the protein. Real-time PCR was then used to assess the effect of METTL16 knockdown on the expression of METTL16 target RNAs. As shown in Fig 5A, 48 hour knockdown of METTL16 caused small changes in expression of a number of RNAs but significantly increased the expression of NT5DC2 mRNA. Interestingly, many of these changes were reduced, lost, or even reversed after 6 days of METTL16 knockdown, and instead we observed decreases in the MALAT1 lncRNA and STUB1 mRNAs (Fig 5B) although it was only with the #7 siRNA that showed greater METTL16 knockdown. No changes were seen in the expression levels of a number of other coding and noncoding RNAs. Consistent with the real-time PCR results, we saw little to no effect of METTL16 knockdown on the protein expression of any of the targets tested (Fig 6).

## Discussion

Overall, our work provides new insight into both the targets of METTL16 as well as its potential for the regulation of gene expression while also raising some new questions about METTL16’s role in the cell. Through immunoprecipitation we have verified the three most well-defined and researched METTL16 RNA substrates [28–30]. While U6 snRNA was the preferred target of FLAG-METTL16, it appears that MAT2A may be the preferred endogenous target in HEK293Ts. We also verified the first identified METTL16 target, lncRNA MALAT1 [30]. Previous reports have found METTL16 bound to ribosomal RNA [28], and our data suggests that 18S rRNA is the preferred target. However, recent studies have identified ZCCHC4 and METTL5 as the 28S and 18S rRNA m6A methyltransferases respectively [44, 45]. This raises the question as to whether the rRNAs are indeed specific binding targets of METTL16, and if they are, what role might METTL16 be playing.

While MAT2A was clearly the preferred mRNA binding target of METTL16 in our system, we were also able to confirm a number of other mRNA binding targets including RBM3, STUB1, and NT5DC2 identified in previous studies [28, 29]. In addition, our studies have identified several novel mRNAs including β2M, HIF-1α and MYC which also appear to be bound by METTL16. However, our long-term knockdown studies failed to reveal any significant expression changes at either the RNA or protein level of these targets. The question of whether these mRNAs are regulated in some way by METTL16 methylation or even METTL16 protein binding remains to be determined.

The change in target preference that we saw between exogenous and endogenous METTL16 immunoprecipitations made us question whether expression levels might impact METTL16 cellular localization, and in turn, modulate its binding preferences. Biochemical fractionation of HEK293T cells suggests that a majority of METTL16 protein is localized to the cytoplasm with lesser amounts of protein found in the nucleus. This contradicts previous characterizations of METTL16 as a predominately nuclear m6A methyltransferase [28–30, 41]. We also observed cytoplasmic METTL16 localization in a number of other cell types representing normal cells as well as different stages of cancer suggesting that this may be a more general finding relevant to all cell types.

Our attempts to verify the biochemical fractionation results with immunohistochemistry were unsuccessful due to non-specific binding of the antibodies tested. This was quite surprising as the antibodies were verified to recognize METTL16 in western blotting as confirmed by loss of signal with long-term siRNA treatment. However, when siRNA knockdown cells were probed for METTL16 by immunohistochemistry, similar signal intensities and localization were seen, suggesting that in immunohistochemistry the antibodies were recognizing other antigens. This explanation is supported by the fact that non-specific bands were also observed in the Western blotting and may be contributing to this erroneous signal. Thus, we feel the immunohistochemistry results are suspect until a specific antibody can be verified.

While it is clear from our fractionation data that METTL16 can be found in the cytoplasm of cells, all of its currently reported activities take place in the nucleus [29, 32, 41]. Splicing of MAT2A is clearly a nuclear activity, as is U6 snRNA processing. Small amounts of U6 snRNA have been found in the cytoplasm but it is not clear if this is a widespread phenomenon [46, 47]. Finally, to date, the majority of METTL16-mediated methylations have been identified in introns [28], which again suggests a nuclear role for the protein. While we have clearly shown METTL16 protein in the cytoplasm of cells, the role it plays there is unclear. It is interesting that in our study, all of our primers were directed to mature mRNA and do not amplify pre-mRNA, which suggests that METTL16 can bind to mature mRNA, although we did not investigate its methylation of those mRNA.

METTL16 is essential for mammalian life based on our (and others) attempts at creating METTL16 knockout cell lines via CRISPR and knockout studies in mice embryos [29, 31]. This observation is also supported by global screens for essential genes that have identified METTL16 [36–40]. The reason for its essentiality is unclear, but it has been implicated in two major roles in cellular development and survival. It is clear that METTL16’s regulation of MAT2A mRNA levels via the SAM synthetase pathway affects developmental events [31]. METTL16 knockout downregulates MAT2A mRNA levels, which likely leads to the production of blastocysts that are not capable of further development. It is also possible that METTL16-mediated m6A methylation of U6 snRNA is important for proper functionality of the spliceosome complex, and the lack of this modification could have global effects on splicing that result in production of incorrect proteins and eventual cell death. It is known that the methylated adenosine (A43) is essential for U6 snRNAs function in yeast [48], however, widespread changes in splicing were not observed in the knockout mouse studies [31], raising the question of whether the methylation of this residue of U6 snRNA impacts splicing in any appreciable way.

Given that METTL16 appears to be essential, conditional knockout/knockdown models will need to be developed to better investigate the effects of METTL16 methylation on mRNA stability, translation, as well as splicing via U6. Of particular interest will be determining whether there are differences between METTL16-mediated methylation and METTL3-mediated methylation, particularly in the RNA binding proteins which recognize the methylation and/or the functional consequences of methylation. In the future, it will be interesting to create METTL16 variants with localization sequences that force the protein to either the nucleus or cytoplasm. These variants can be used to determine the binding preferences of METTL16 based on its localization, which may explain the differences we have seen in this study.

## Materials and methods

### Tissue culture

HEK293T, HEK293, HELA, CCD34LU and NCI-H1299 cells were obtained directly from ATCC. MCF10-A, MCF10-AT1, and MCF10-Ca1h were obtained from Barbara Ann Karmanos Cancer Center. Cells were routinely cultured at 37˚C, 5% CO_2_. HEK293T, HEK293, and HELA cells were maintained in DMEM with 4g/L glucose, 10% FBS, 2 mM L-glutamine, and 1X Pen/Strep. CCD34LU cells were maintained in EMEM with 10% FBS, and 1X Pen/Strep. NCI-H1299 cells were maintained in RPMI-1640 with 4g/L glucose with 10% FBS, 2 mM L-glutamine, and 1X Pen/Strep. MCF10-A, MCF-AT1, and MCF10-Ca1h cells were maintained in a 50/50 mix of DMEM/F12 with 5% horse serum, 1mM CaCl_2_, and 1X Pen/Strep, 10 μg/ml insulin, 20 ng/ml EGF, 0.5 μg/ml Hydrocortisone, and 0.1 μg/ml cholera enterotoxin.

### Knockdown and overexpression of METTL16

For knockdown, HEK293T cells were transfected with 20 μM siRNA (Life Technology Silencer Select) targeting METTL16 (siRNA ID# S35507 or S35509) or Negative Control #1 (Catalog # AM4635) siRNA with Lipofectamine 2000 (Thermo Fisher) according to manufacturer’s directions. For long-term knockdown, cells were transfected and allowed to recover for 48 hours. Cells were lifted, counted and replated for 2^nd^ round of transfection while the remainder of the cells were harvested for RNA and protein. The following day cells were transfected again and allowed to recover for 3 more days before final harvesting (6 days knockdown total). For overexpression, 2 μg of plasmid expressing a FLAG-tagged METTL16 (Origene: RC208648) or GFP were used. Cells were transfected for 72 hours before harvesting to allow for sufficient overexpression of METTL16.

### Western blots

Whole-cell lysates were prepared in whole-cell extract buffer (WCEB: 50 mM Tris pH 7.4, 150 mM NaCl, 5 mM EDTA, 0.1%SDS, and complete protease inhibitor [Promega]). After sonication, equal amounts of protein (30–50 μg) were electrophoresed on a mini-PROTEAN any KD acrylamide gel (Bio-Rad Laboratories) and transferred to Hybond ECL nitrocellulose (GE Healthcare). The blot was blocked with 5% non-fat dry milk (LabScientific) in Tris-buffered saline with 0.1% Tween 20 (TBST) for 1 hour at room temperature, followed by primary antibody incubation in blocking buffer overnight at 4°C. After washing extensively with TBST, blots were incubated for 1–2 hour at room temperature with appropriate HRP-linked secondary antibody (GE Healthcare), washed again with TBST, developed using Bio-Rad Clarity Western ECL Substrate (Bio-Rad Laboratories), and imaged via MYECL Imager (Thermo Scientific). Antibody details including working dilutions can be found in S1 Table.

### RNA extraction

Trizol (Life Technologies) was used for all RNA extractions according to the manufacturer’s protocol. For RNA extraction from ribonucleoprotein immunoprecipitations (RIP), GlycoBlue (Life Technologies) was added as a carrier during the precipitation step. RNA quality and quantity were determined via NanoDrop 1000 (ThermoFisher Scientific).

### PCR

Reverse transcription was performed on 1 μg of total RNA in a 20 μl reaction with the iScript cDNA synthesis kit (Bio-Rad Laboratories,170–8891). Quantitative real-time PCR was performed using a Roche Lightcycler 96 with Fast Start Essential DNA Green (Roche Diagnostics Corporation, 06-924-204-001) and primers from Integrated DNA Technologies, Inc. Primer efficiency was verified to be over 95% for all primer sets used. Quantification of mRNA was carried out via ΔΔCT analysis using GAPDH mRNA and the respective control condition for normalization. All real-time PCR primer sets were designed so the products would span at least one intron (>1kb when possible) to prevent detection of the pre-mRNA and/or DNA, and amplification of a single product was confirmed by agarose gel visualization and/or melting curve analysis (See S2 Table for sequences).

### FLAG immunoprecipitation of METTL16

Beads labeled with FLAG Antibody (Sigma) were washed and resuspended in NT2 buffer (50 mM Tris-HCl (pH 7.4), 150 mM NaCl, 1 mM MgCl_2_, 0.05% NP40) supplemented with 1 mM DTT, 100 units/ml RNase Out and 20mM EDTA. Cells were harvested in Polysome Lysis Buffer (PLB; (100 mM KCl, 5 mM MgCl_2_, 10 mM HEPES (pH 7.0), 0.5% NP40, 1 mM DTT, 100 units/ml RNase Out, with Protease inhibitor cocktail)) and equal amounts of lysate were added to each IP reaction and tumbled for 4 hours at 4°C. After washing 5 times with NT2, beads were resuspended in Trizol for RNA or WCEB with protease inhibitors (PI) for protein. Relative and fold enrichment of RNA in the IP was determined as indicated in the results section.

### Immunoprecipitation of endogenous METTL16

Cells were harvested in PLB. Beads labeled with METTL16 Antibody (Bethyl Laboratories) or normal rabbit serum (NRS) were washed and resuspended in NT2 supplemented with 1 mM DTT, 100 units/ml RNase Out and 20mM EDTA. Equal amounts of lysate were added to each IP and tumbled for 4 hours at 4°C. After washing, beads were lysed in Trizol (RNA) or WCEB (protein). Relative and fold enrichment was determined as indicated in the results section.

### Cell fractionation

For total extracts, 10% of the cells were lysed in WCEB with Pierce protease inhibitor cocktail (PI; Thermo Scientific). The remaining cells were resuspended in hypotonic buffer (10 mM NaCl, 10 mM Tris-HCl, pH 7.4, 1.5 mM MgCl2 with PI) and incubated on ice for 5 minutes before freezing at -80°C. After thawing on ice, lysis was achieved by vortexing for 2–3 seconds and nuclei were pelleted at 1,000 xG for 5 minutes at 4°C. The supernatant was removed and stored as cytoplasmic fraction. The pellet was then resuspended in the above buffer containing 1% NP40 and 0.5% sodium deoxycholate and centrifuging at 1,000 xG for 5 minutes at 4°C. The supernatant after centrifugation was designated the soluble nuclear fraction. The remaining insoluble nuclear pellet was sonicated in WCEB with PI. Equal volumes of each fraction were subjected to western blotting as described above. MyImage Analysis (Thermo Scientific) was used to quantify the bands in the cytoplasmic, soluble nuclear and insoluble nuclear fractions. Data were expressed as a percentage of the total protein (summed from the three fractions) found in each fraction.

### Immunohistochemistry

HEK293T cells with or without 6 days of siRNA treatment (as described above) were fixed in either ice cold 4% paraformaldehyde or 95% methanol/5% acetic acid for 15mins. Paraformaldehyde-treated cells were permeabilized with 0.1% Triton in PBS for 10 minutes. After washing with PBS, cells were blocked with 1% BSA in PBS at 4°C for at least 1 hour. Primary antibody incubation was performed for at least 16 hours in blocking buffer, followed by extensive washing in PBS. Secondary antibody incubation was performed for 1.5 hours in blocking buffer at room temperature in the dark, followed by extensive washing with PBS. Cells were then counterstained with DAPI before being imaged on an Olympus IX73 inverted compound microscope. Images were captured with Olympus cellSens software using default settings. Briefly, a random field of Neg siRNA treated cells were imaged for phase contrast, FITC, and DAPI and the exposure times for the FITC and DAPI stains were then used for subsequent images of the siRNA treated cells to allow for comparison between the two conditions. A secondary antibody-only control was also imaged with this same exposure. All individual images were converted to lossless JPEGs without any modifications from the original .vsi file. The “Combine Channels” option in the cellSens software was used to create the FITC/DAPI overlays. For clearer visualization and background reduction, the overlays were subjected to the “Optimize Contrast” option in the cellSens software.

### Statistical analysis

All experiments were performed on at least three separate occasions to generate biological replicates unless otherwise indicated. For the immunoprecipitations, statistical significance was calculated by a two-tailed, paired Student’s t-test comparing the METTL16 IP to the GFP or NRS IP. Outliers were identified utilizing the Grubb’s test but only one outlier was ever removed for a given RNA target. For the real time analysis of the siRNA knockdown experiments, a one-way ANOVA with post-hoc Tukey HSD test was run for each RNA target comparing all six conditions. For all experiments, a P-value below 0.05 was defined as statistically significant, while a P-value less than 0.1 was considered reportable.

## Supporting information

S1 File Raw Western blot images. (PDF)

S1 Fig Validation of HPA020352 METTL16 antibody. (A) Methanol fixed HEK293T cells subjected to immunohistochemistry with METTL16 antibody and DAPI nuclear stain. (B) HEK293T cells were treated for 6 days with either a negative control siRNA (Neg) or METTL16-specific siRNAs. Western blotting indicated substantial METTL16 knockdown (similar to Fig 6) with additional non-specific background bands. Immunohistochemistry on methanol fixed cells from the same experiment showed similar staining in both location and intensity despite METTL16 knockdown suggesting non-specific binding.
(TIF)

S2 Fig Immunohistochemistry with second METTL16 antibody. HEK293T cells were treated for 6 days with either a negative control siRNA (Neg) or METTL16-specific siRNAs. Immunohistochemistry on paraformaldehyde fixed cells with PA5-54185 METTL16 antibody showed similar staining in both location and intensity despite METTL16 knockdown suggesting non-specific binding. DAPI was used to visualize the nucleus.
(TIF)

S1 Table Antibodies used in this study. (DOCX)

S2 Table Real time PCR primers used in this study. (DOCX)
