# Supplementary material for: Characterization of METTL16 as a cytoplasmic RNA binding protein
Source: PLoS One. 2020 Jan 15;15(1):e0227647. doi: 10.1371/journal.pone.0227647 (PMC6961929; doi:10.1371/journal.pone.0227647)

Fig 1A Original Image

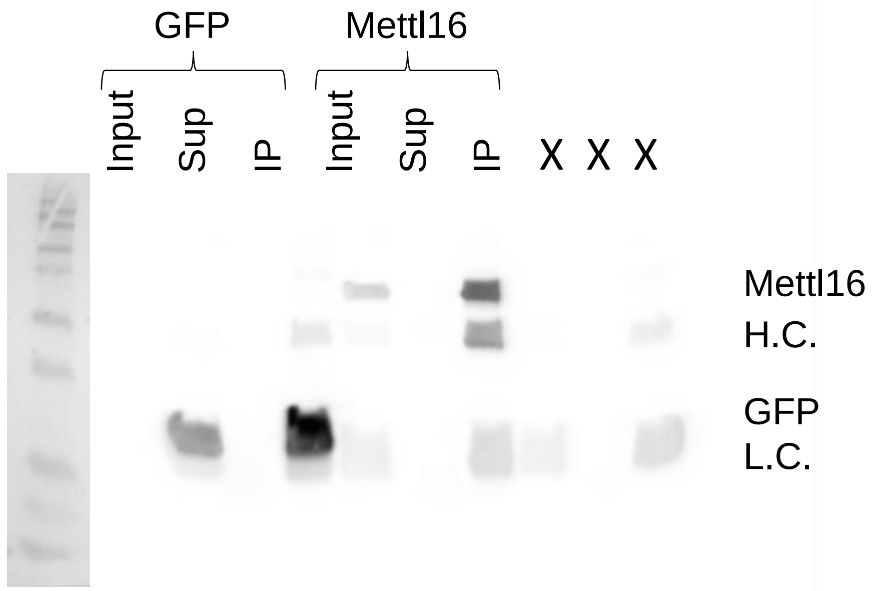

Fig 2A Original Image

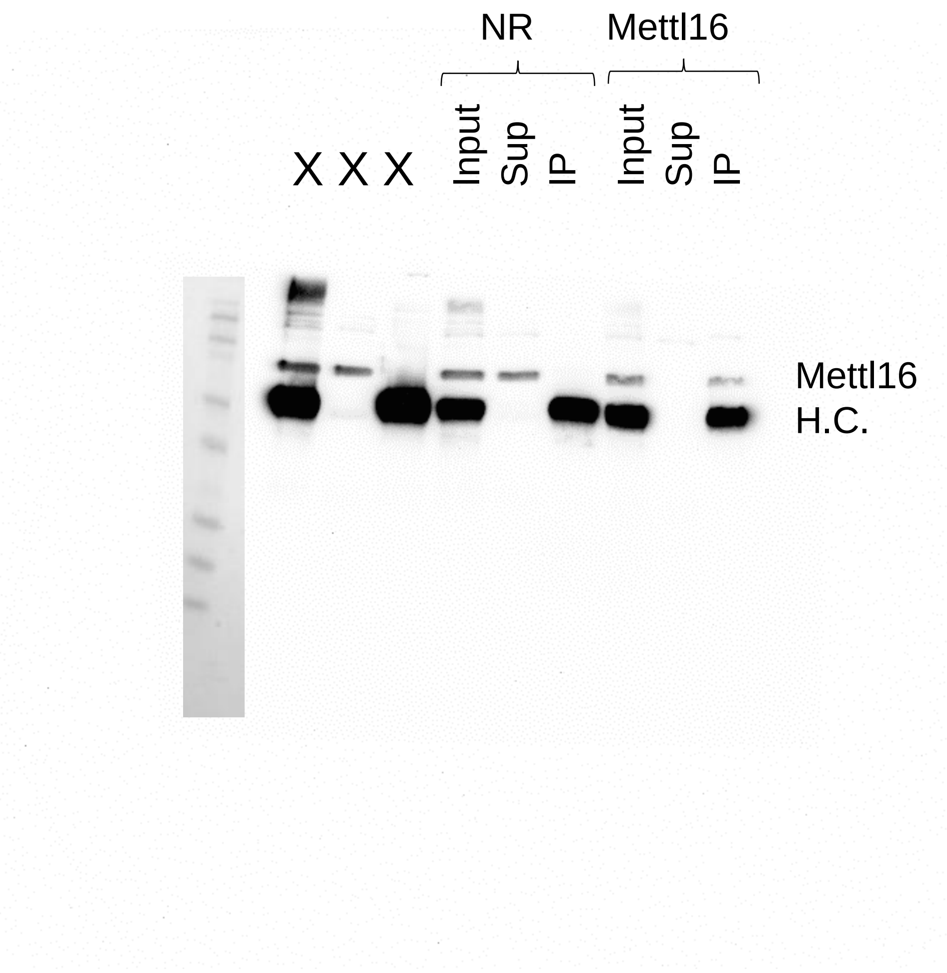

Fig 3A Original Images

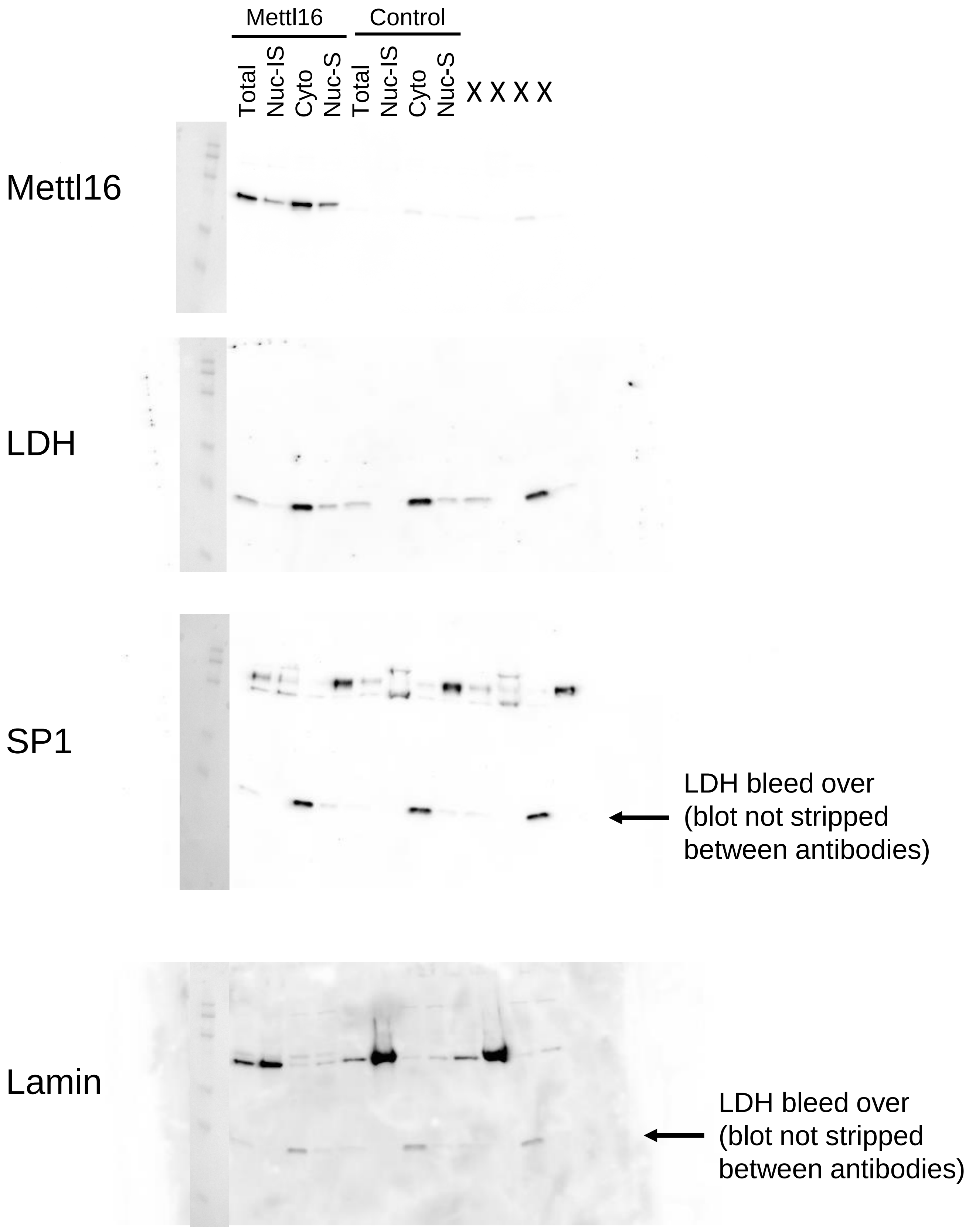

Fig 3B Original Images

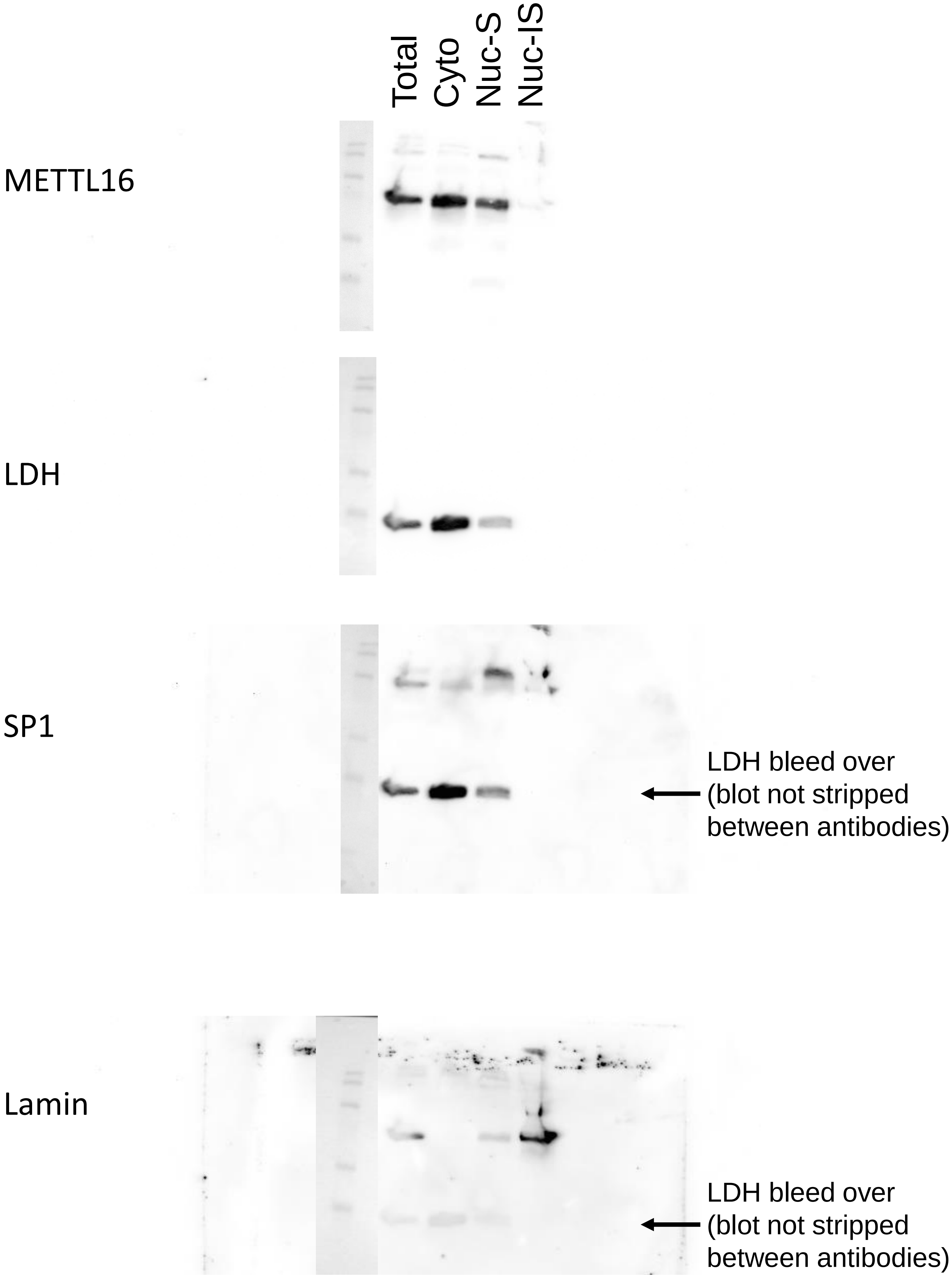

Fig 4A Original Images

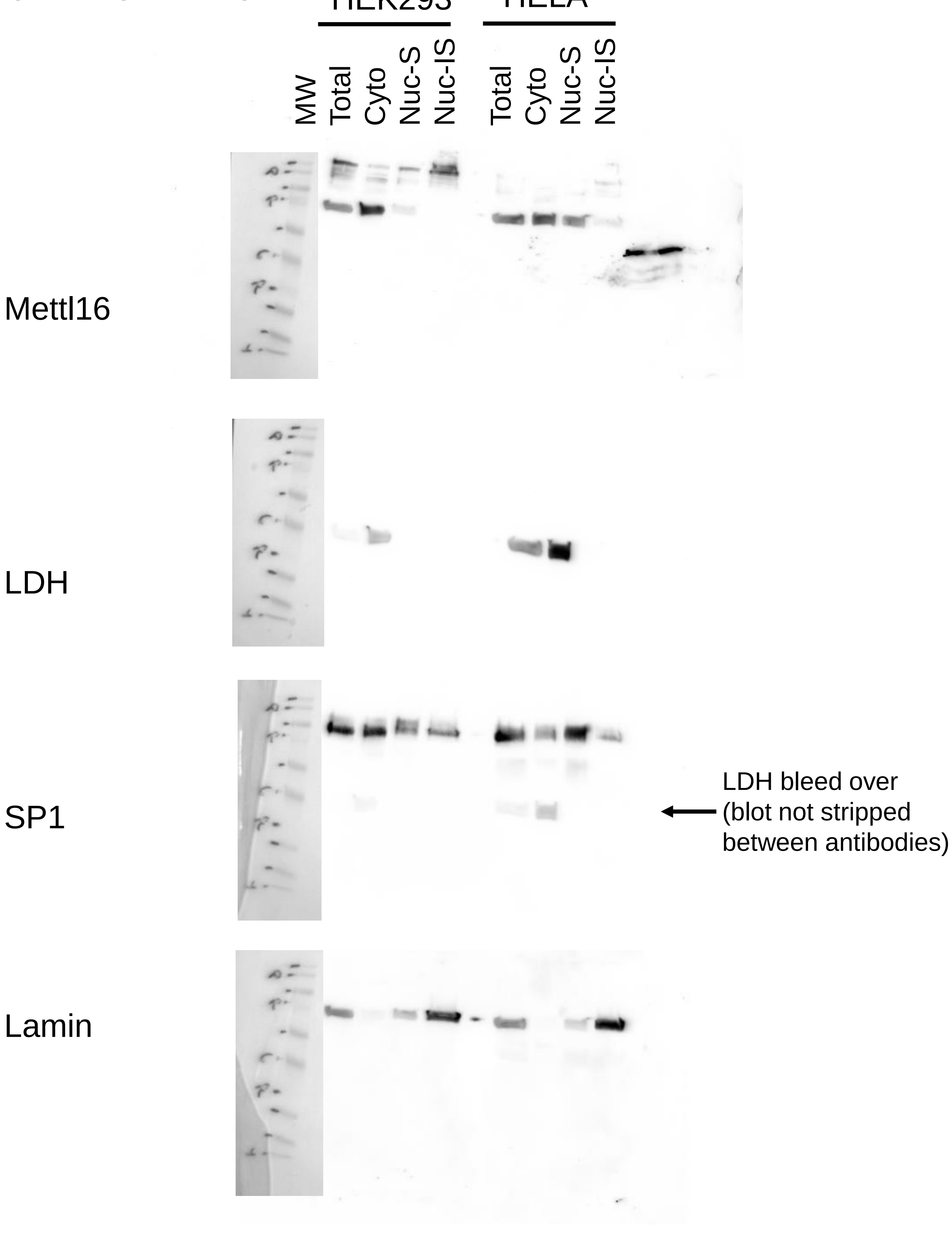

Fig 4B Original Images

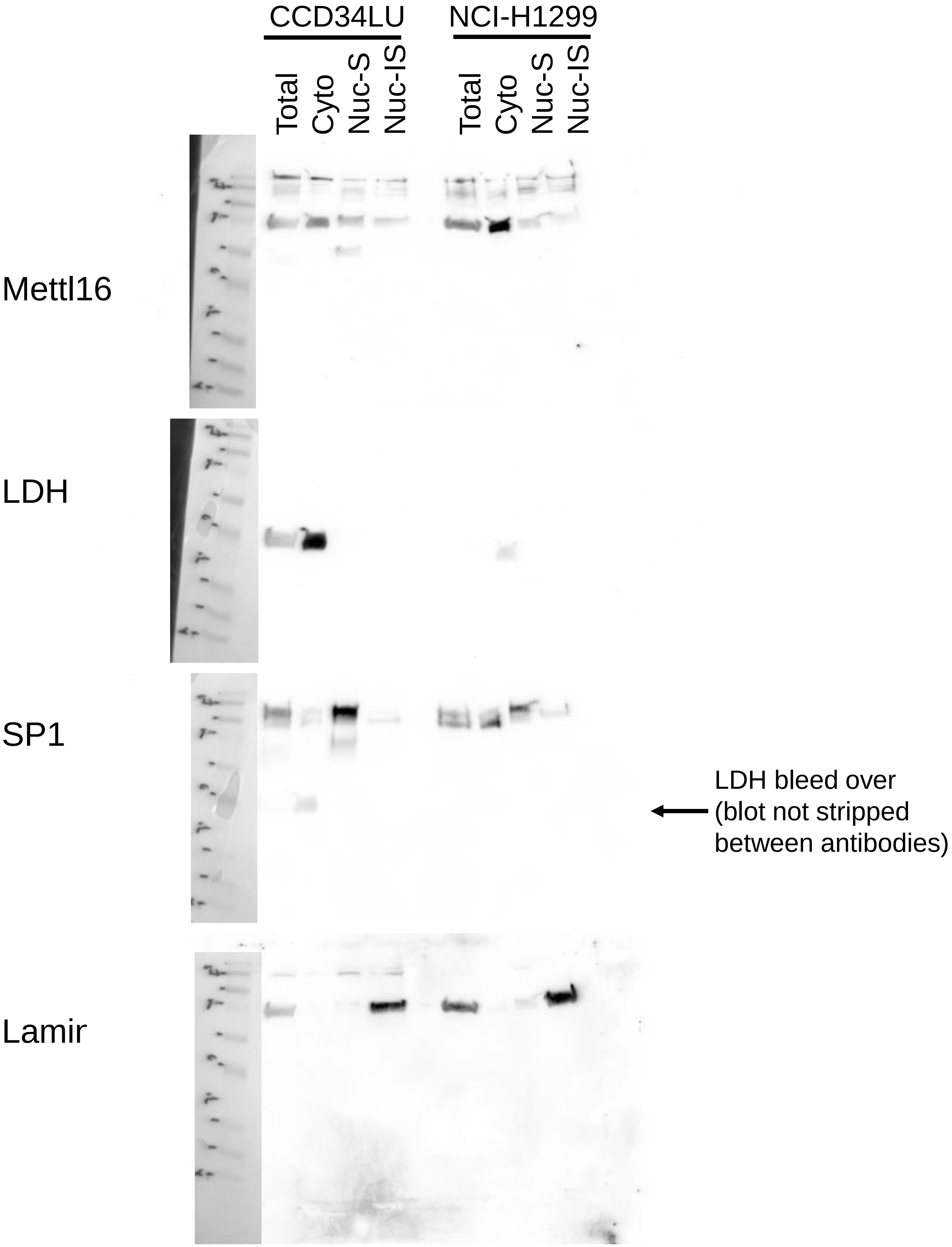

Fig 4C Original Images

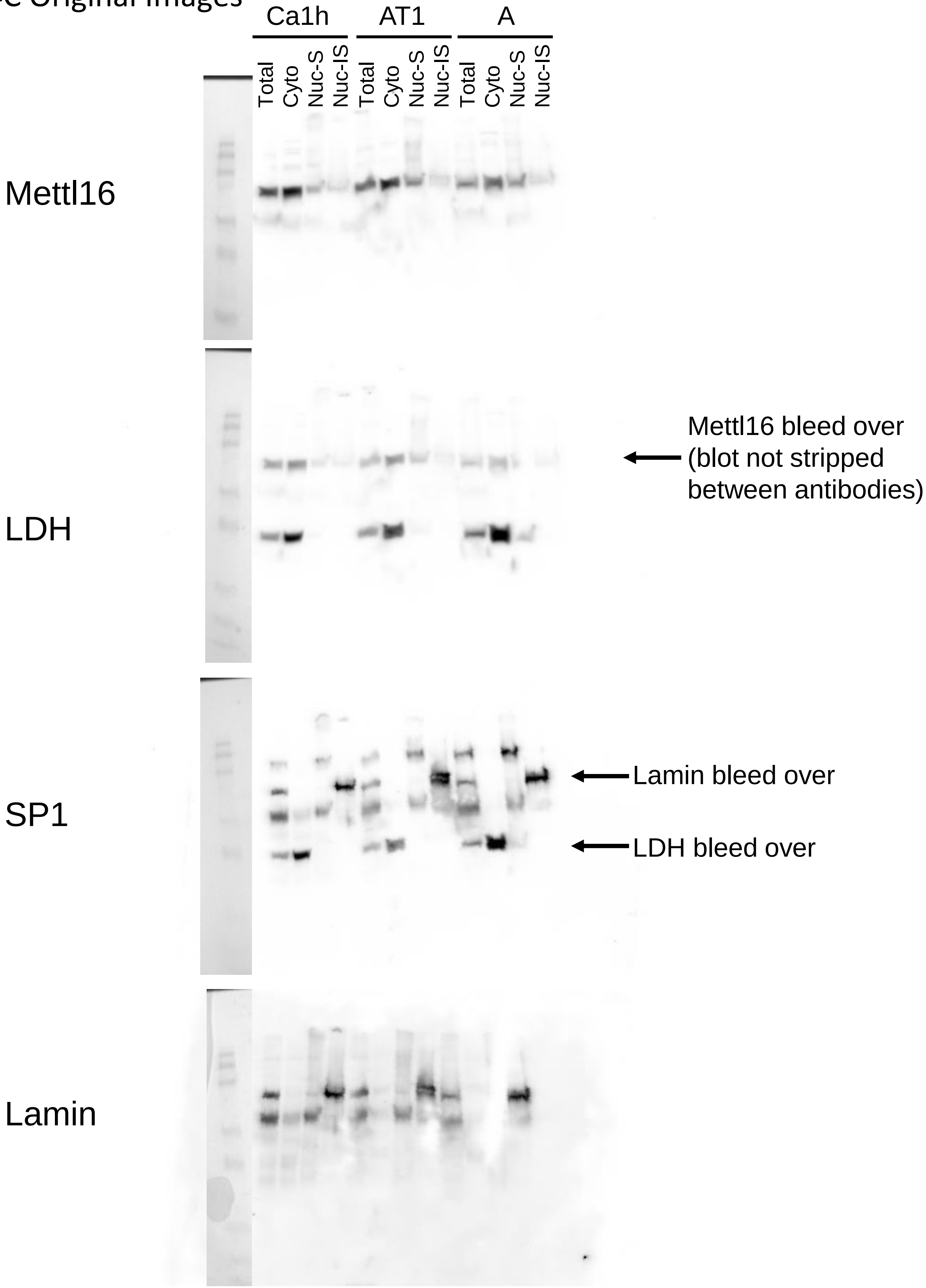

Fig 6 Original Images

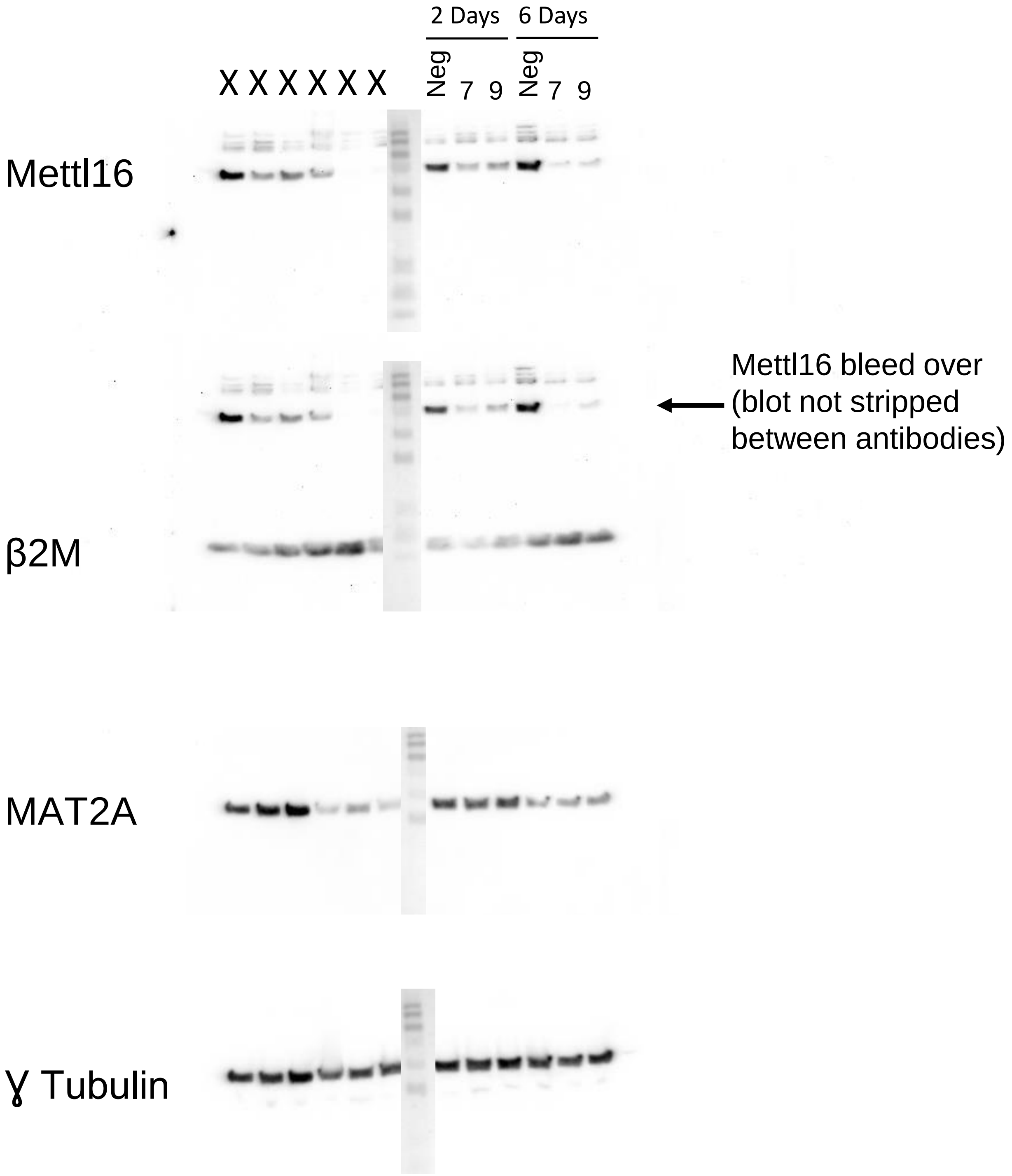

S1 Fig Original Images

Mettl16

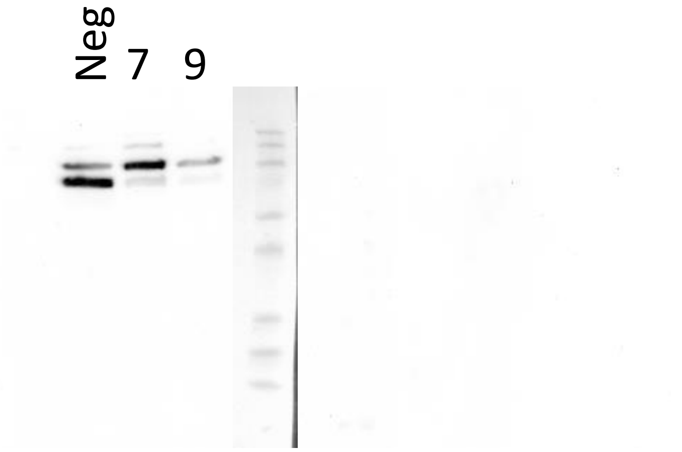

ɣ Tubulin

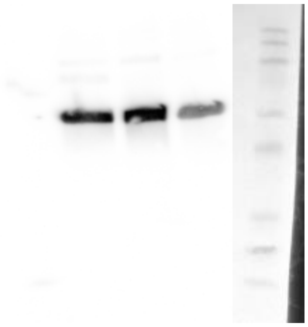

Supplement: S1 File — (PDF) [file pone.0227647.s001.pdf]
