# Supplementary figures and images for: Characterization of METTL16 as a cytoplasmic RNA binding protein
Source: PLoS One. 2020 Jan 15;15(1):e0227647. doi: 10.1371/journal.pone.0227647 (PMC6961929; doi:10.1371/journal.pone.0227647)

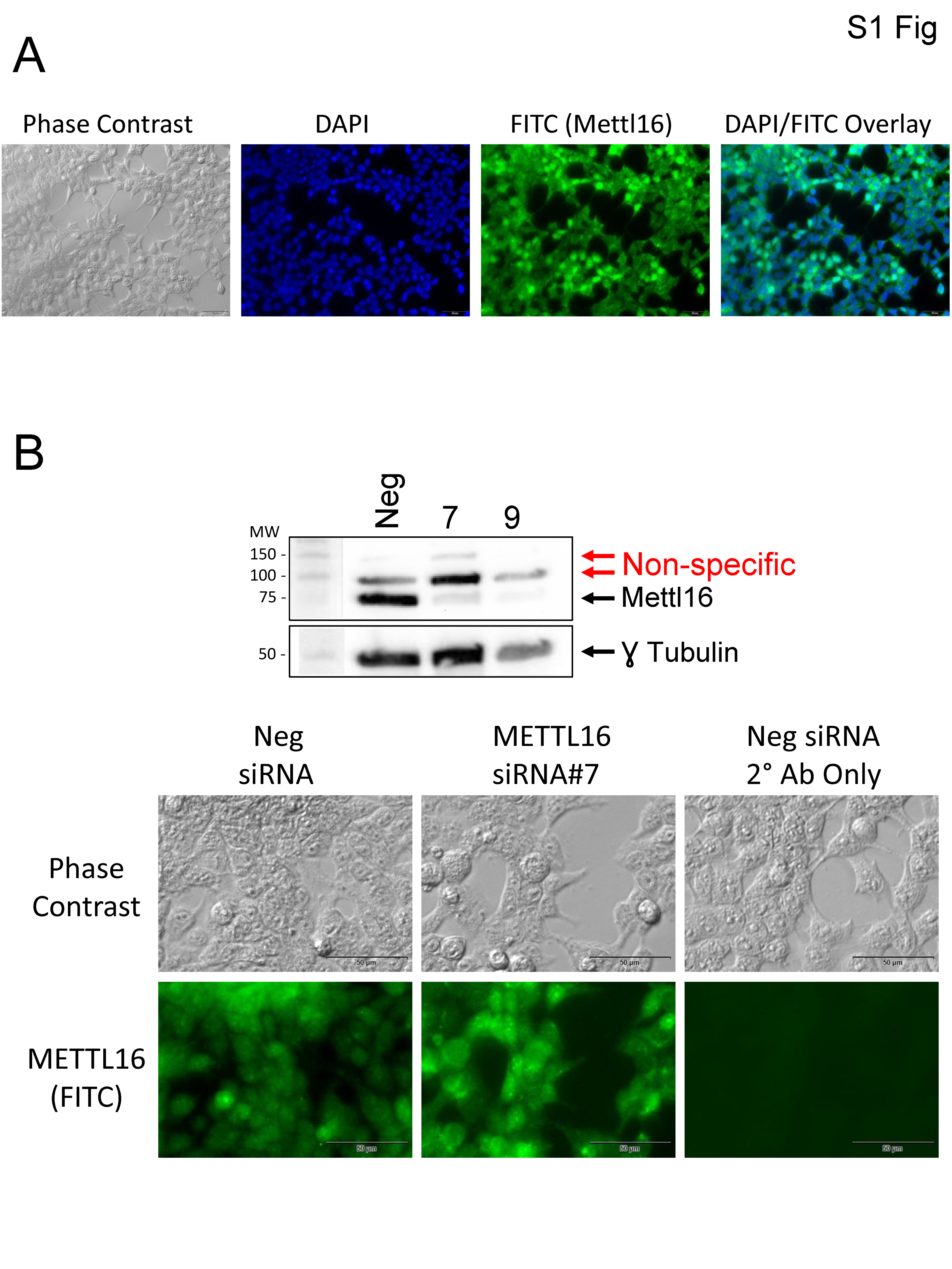

Supplement: S1 Fig — (A) Methanol fixed HEK293T cells subjected to immunohistochemistry with METTL16 antibody and DAPI nuclear stain. (B) HEK293T cells were treated for 6 days with either a negative control siRNA (Neg) or METTL16-specific siRNAs. Western blotting indicated substantial METTL16 knockdown (similar to Fig 6) with additional non-specific background bands. Immunohistochemistry on methanol fixed cells from the same experiment showed similar staining in both location and intensity despite METTL16 knockdown suggesting non-specific binding. (TIF) [file pone.0227647.s002.tif]

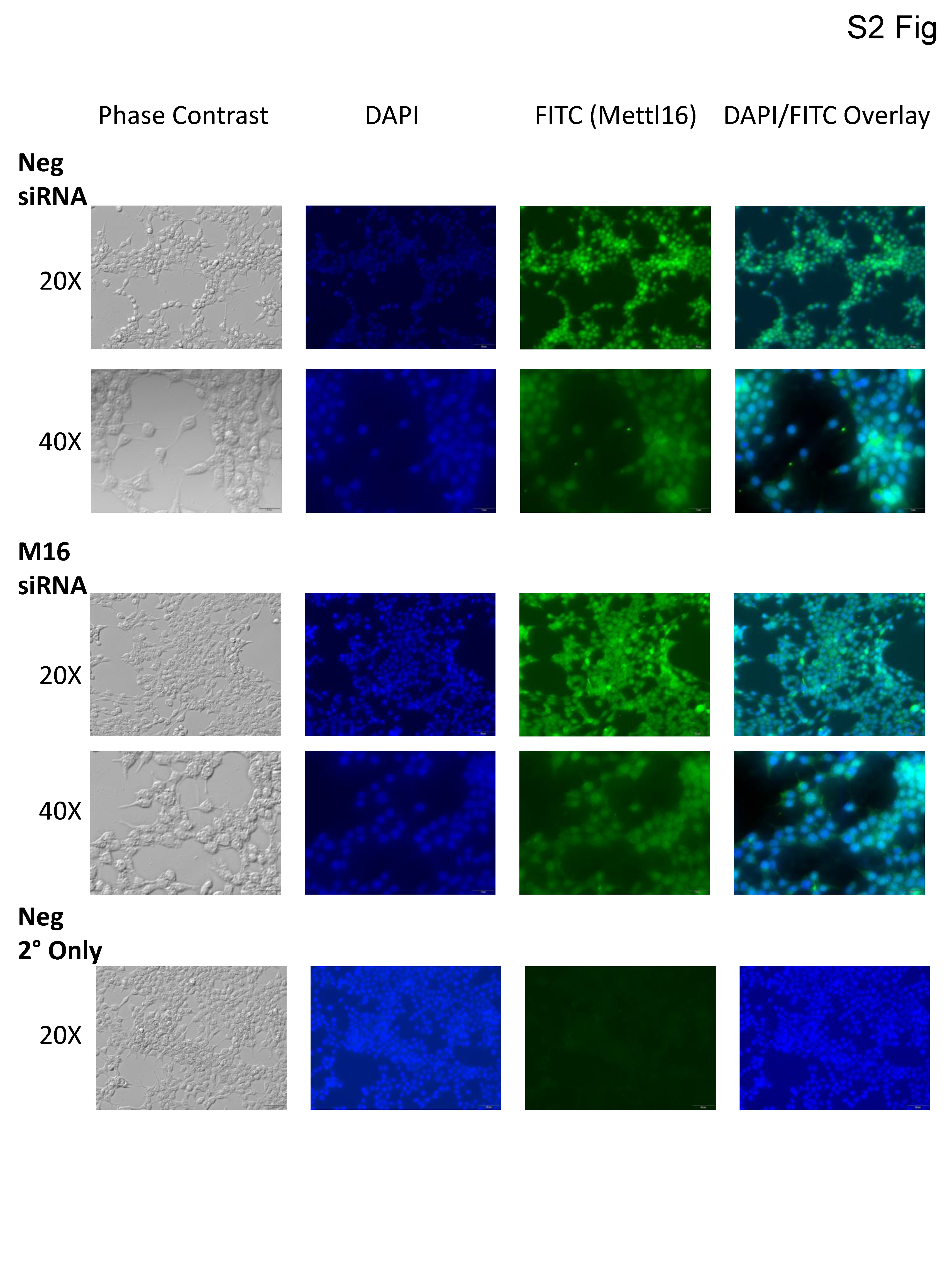

Supplement: S2 Fig — HEK293T cells were treated for 6 days with either a negative control siRNA (Neg) or METTL16-specific siRNAs. Immunohistochemistry on paraformaldehyde fixed cells with PA5-54185 METTL16 antibody showed similar staining in both location and intensity despite METTL16 knockdown suggesting non-specific binding. DAPI was used to visualize the nucleus. (TIF) [file pone.0227647.s003.tif]
