# Supplementary material for: Characterization of METTL16 as a cytoplasmic RNA binding protein
Source: PLoS One. 2020 Jan 15;15(1):e0227647. doi: 10.1371/journal.pone.0227647 (PMC6961929; doi:10.1371/journal.pone.0227647)
Supplement: S1 Table — (DOCX) [file pone.0227647.s004.docx]

**Supplemental Table 1: Antibodies Used**

| **Antibody** | **Catalog #** | **Vendor** | **Dilution** |
| --- | --- | --- | --- |
| β-2-Microglobulin | 12851 | Cell Signaling | 1:1,000 (WB) |
| FLAG | MA1-91878 | Thermo Fisher | 1:1,000 (WB) |
| Lamin B | SC-6216 | Santa Cruz | 1:200 (WB) |
| LDH | SC-133123 | Santa Cruz | 1:1,000 (WB) |
| MAT2A | SC-166452 | Santa Cruz | 1:200 (WB) |
| Mettl16 | A304-192A | Bethyl Labs | 1:1,000 (WB) |
| Mettl16 | HPA020352 | Millipore Sigma | 1:1000 (WB)  1:200 (IHC) |
| Mettl16 | PA5-54185 | Invitrogen | 1:200 (IHC) |
| SP1 | MA5-27783 | Invitrogen | 1:1000 (WB) |
| Ɣ-Tubulin | MA1-850 | Thermo Fisher | 1:1,000 (WB) |
