# Supplementary material for: Characterization of METTL16 as a cytoplasmic RNA binding protein
Source: PLoS One. 2020 Jan 15;15(1):e0227647. doi: 10.1371/journal.pone.0227647 (PMC6961929; doi:10.1371/journal.pone.0227647)
Supplement: S2 Table — (DOCX) [file pone.0227647.s005.docx]

**S2 Table: PCR Primers Used**

| Gene | Forward (5'-3') | Reverse (5'-3') |
| --- | --- | --- |
| 18s rRNA | CTGAGAAACGGCTACCACATC | GCCTCGAAAGAGTCCTGTATTG |
| 28s rRNA | GGGTGGTAAACTCCATCTAAGG | GCCCTCTTGAACTCTCTCTTC |
| 5s rRNA | CGTCTGATCTCGGAAGCTAAG | CCTACAGCACCCGGTATTC |
| β2M | AGATGTCTCGCTCCGTGGCCTTA | TGTCGGATGGATGAAACCCAGACA |
| GapDH | AAGGTCGGAGTCAACGGATTTGGT | AGCCTTGACGGTGCCATGGAATTT |
| HIF1α | CCGAATTGATGGGATATGAGCCAG | TTGGCAAGCATCCTGTACTGTCCT |
| MALAT1 | GAATTGCGTCATTTAAAGCCTAGTT | GTTTCATCCTACCACTCCCAATTAAT |
| MAT2A | CTGCTGTTGACTACCAGAAAGT | GCTACCAGCACGTTACAAGT |
| METTL3 | AGCCTTCTGAACCAACAGTCC | CCGACCTCGAGAGCGAAAT |
| METTL16 | GGCAGAAGGAGGTGAATTAGAG | TTCCCAGCATGCAGCTATAC |
| Myc | TCCTCGGATTCTCTGCTCTCCT | AGAAGGTGATCCAGACTCTGACCT |
| NT5DC2 | GATGAGAAGGGCTCACTTCAG | CCATTCCGTCAAGCGTAAGA |
| RBM3 | TTCATCACCTTCACCAACCC | ATCTGACGACCATCCAGAGA |
| STUB1 | GGCCAAGCACGACAAGTA | GATCTTGCCACACAGGTAGTC |
| U1 | CCATGATCACGAAGGTGGTTT | ATGCAGTCGAGTTTCCCACAT |
| U2 | TTCTCGGCCTTTTGGCTAAG | CTCCCTGCTCCAAAAATCCA |
| U4 | GCCAATGAGGTTTATCCGAGG | TCAAAAATTGCCAATGCCG |
| U6 | CTCGCTTCGGCAGCACA | AACGCTTCACGAATTTGCGT |
